# Synthesis, purification and crystallization of a putative critical bulge of HAR1 RNA

**DOI:** 10.1371/journal.pone.0225029

**Published:** 2019-11-08

**Authors:** Monica R. Lares

**Affiliations:** Chemistry Department, Sonoma State University, Rohnert Park, California, United States of America; Virginia Commonwealth University, UNITED STATES

## Abstract

Non-coding RNAs have raised a lot of interest because of their capabilities to perform enzymatic reactions and regulate gene expression in various ways. Human Accelerated Region 1 (HAR1) has been identified during the search for highly conserved regions in mammalian genomes, over one hundred base pairs long, and with high rates of substitution in the human genome. Its potential for coding for a protein is very minimal. However, the HAR1 transcript has been computationally predicted to have a stable secondary structure. Previous structure-probing experiments have suggested that the majority of differences between human and chimp constructs are in helices, designated C and D. For this reason, a 47nt construct consisting of the C and D helices along with two additional C-G pairs was synthesized, purified, and crystallized, and its x-ray structure is reported in this study. The final structure is an artificial dimer, with a bulge that forms different conformations on each monomer. This bulge has been observed in predicted secondary structures, footprinting assays, enzymatic degradation assays, NMR studies, *in silico* studies, and in this crystalized dimer structure. It is proposed that the HAR1 transcript is a non-coding RNA that interacts with an unknown binding partner responsible for brain development through this inherent structural motif of bulged adenosines.

## Introduction

Human Accelerated Region 1 (HAR1) was identified as a non-coding RNA that has a putative role in mammalian brain development [1]. HAR1 is strongly and specifically expressed in the neocortex of the developing fetus at seven to nine gestational weeks. The neocortex is the structure that provides the basis of human mental capacity and uniqueness [2]. HAR1 is suggested to be selectively expressed in Cajal-Retzius neuronal cells [1]. Cajal-Retzius neurons are responsible for early neuronal circuitry in the developing brain and for the expression of genes involved in cerebral development.

HAR1 exhibits a significant rate of substitution in humans and is thus a part of a group of molecules named human accelerated regions (HARs). HAR1 has 18 substitutions in a region that would be expected to only have had 0.27 substitutions since the last common ancestor between humans and chimpanzees. The HAR1 region in chimpanzees has only two substitutions compared to chickens. HAR1 has not been detected in frogs, fish, or any invertebrate genomes. Resequencing HAR1 in a 24-person diversity panel confirmed that all 18 substitutions are fixed in the human population [1]. All of the human-specific substitutions are from adenosine or thymine bases to cytosine or guanine bases.

The HAR1 RNA transcript was predicted to form a stable secondary structure by a computer program that considers RNA evolution, Evofold [3]. The structure predicted by Evofold was published by Pollard, and the helices were designated A-E. Beniaminov *et al*., using three enzymatic probes and three chemical probes, found human and chimpanzee HAR1 RNA secondary structures to differ from those previously published as well as to dramatically differ from each other [4]. NMR studies of portions of HAR1 agreed with Beniaminov *et al*.*’s* structures and showed that human HAR1 RNA is more dynamic compared to that of chimpanzee [5]. *In silico* studies have been carried out to uncover the structural evolution of HAR1 RNA and have pointed to a more stable secondary structure in the human construct [6]. However, the exact structure and function of HAR1 remains elusive.

Pollard’s structure-probing experiments suggested that the majority of differences between the human and chimp constructs were in the C and D helices. For this reason, a 47nt construct consisting of the C and D helices, along with two additional C-G pairs to ensure strong annealing between the two ends to aid in the formation of the helices, was synthesized, purified, and crystallized, and its structure is reported in this study.

## Material and methods

### Macromolecular production

Pollard observed strong modification with DMS of A70, which is present in the predicted bulge connecting the C and D helices. The desired 47nt HAR1 RNA construct for this study, consisting of the C and D helices, was *in vitro* transcribed from a 67-nucleotide DNA template containing a double-stranded T7 polymerase promoter. The DNA template was synthesized using an Expedite 8900 instrument. Two guanosines were added to the 5' end, and two cytosines were added to the 3' end, in order to ensure strong annealing between the two ends to aid in the formation of the C and D helices. A 2'-O-methyl modification was added to the second-to-last base in order to reduce the production of the n+1 product [7]. The phosphoramidites used for the *in vitro* synthesis of the DNA templates were purchased from Glen Research.

This DNA template was then used to carry out a T7 *in vitro* transcription, which yielded the desired 47nt RNA construct. The RNA was purified using a 12% polyacrylamide gel. The RNA was resuspended in 2mL of water and desalted using a Centricon filtering device (YM-3). After 12mL of water was passed through the Centricon, the volume was reduced until a sample with a concentration of 10mg/mL was achieved. The purity of the RNA was verified on an analytical 12% acrylamide gel.

### Crystallization

Hanging drop vapor diffusion was used to crystallize the HAR1 RNA. The RNA was folded by mixing water with the RNA to reach the concentrations tested (2, 4, 5, 6, 8, 10mg/mL) and then heated to 70°C for three minutes, after which it was cooled to room temperature; then, 10X crystallization buffer was added (500mM Tris-HCl pH 7.5, 1M KCl, 50 mM MgCl_2_). The hanging drop was made from 2μL of the prepared RNA and 2μL of the screening solution (Natrix Screen, Crystal Screen 1, and Crystal Screen 2 from Hampton Research were all tested). One milliliter of the screening solution was placed in the reservoir, and the tray was set in a 22°C incubator.

Optimizing screens were set up by hand with the various concentrations of RNA. For each cover slip, two drops were prepared: one with crystallization buffer and one without. These drops were allowed to equilibrate for two days. A sterile whisker was used to touch the crystalline material produced, then streaked through freshly equilibrated drops. Large needle-like crystals grew in a solution of 0.1M Na HEPES pH 7.5 and 1.4M Na citrate tribasic dihydrate within two weeks, which were then used in the data collection.

There was no need for further cryoprotectant, as this solution contained enough salt for a glass-like freeze of the liquid surrounding the crystal. Once it was verified that a crystal was in the loop, it was immediately submerged in a dewar of liquid nitrogen and stored until it was needed for data collection.

### Data collection and processing

Synchrotron data were collected at the Stanford Synchrotron Radiation Lightsource (SSRL) on beamline 9–2. Parameters for data collection were as follows: the wavelength was 0.85565 Å, the detector distance was 300mm, the beam stop was set to 40.005 mm, an image was collected every one degree for 20 seconds, and 360° of data were collected. *MOSFLM* [8] was used to process all image files (Table 1). The space group was determined to be P2_1_, and the mosaicity was averaged to 1.28. This file was then scaled, truncated, and sorted by software from *CCP4* (Collaborative Computational Project, Number 4, 1994). Parameters are summarized in Table 1.

**Table 1:** Data collection parameters and processing data.

Diffraction source	SSRL beamline BL9–2
Wavelength (Å)	0.85565
Temperature (K)	80
Detector	Dectris PILATUS3 S 6*M* pixel
Crystal-detector distance (mm)	300
Rotation range per image (°)	**1**
Total rotation range (°)	360
Exposure time per image (s)	20
Space group	*P*2_1_
*a*, *b*, *c* (Å)	61.56, 39.83, 80.97
α, β, γ (°)	90, 111.83, 90
Mosaicity (°)	1.28
Resolution range (Å)	75.00–2.90
Total No. of reflections	29010
No. of unique reflections	8346
Completeness (%)	99.9
Redundancy	3.5
〈*I*/σ(*I*)〉	7.4
*R*_r.i.m._‡	0.050
*R*_p.i.m._	0.086
Overall *B* factor from Wilson plot (Å^2^)	29.7

### Structure solution and refinement

Since this construct is a novel RNA crystal structure without a heavy atom derivative or a known homologous, high-resolution, three-dimensional structure as a model for molecular replacement, the Robertson-Scott method [9] was used to solve the crystallographic phase problem. Once the processing of the native data achieved a satisfactory level using MOSFLM and CCP4 software, this Robertson-Scott method was used to obtain the phase angles. Using typical established RNA structural features as a model, in this case an A-form helix, an iterative molecular-replacement procedure was performed in order to obtain an estimated phase set. Additional features began to emerge within the electron density, indicating that the model had legitimate predictive phasing power and generated an interpretable electron density map. Then, the A-form helix model was completely discarded, but the phases were retained and solvent-flattened with *CNS* [10]. These phases were used to generate an electron density map, which was in turn used for tracing, modeling, and refining the genuine structure. Files were viewed and adjusted in *Coot* [11], processed with *Phaser* [12], and refined with *REFMAC* [13]. This cycle was repeated until a satisfactory model was achieved, one that agreed well with the data. The final refinement parameters are shown in Table 2 (PDB ID: 5UNE; validation report in S1 Fig).

**Table 2:** Structure refinement data.

No. of reflections, working set	8257 (1241)
No. of reflections, test set	812 (136)
Final *R*_cryst_	0.201 (0.325)
Final *R*_free_	0.249 (0.393)
Nucleic acid	2032
Ion	2
Water	15
Total	2049

## Results and discussion

The 47nt construct crystallized into long needle-like crystals. The space group was determined to be P2_1_, and the mosaicity was averaged to 1.28. The Robertson-Scott method [9] was used to solve this novel RNA crystal structure without a heavy atom derivative or a known homologous, high-resolution, three-dimensional structure as a model for molecular replacement. The final unit cell parameters are shown in Table 1.

**Fig 1:**
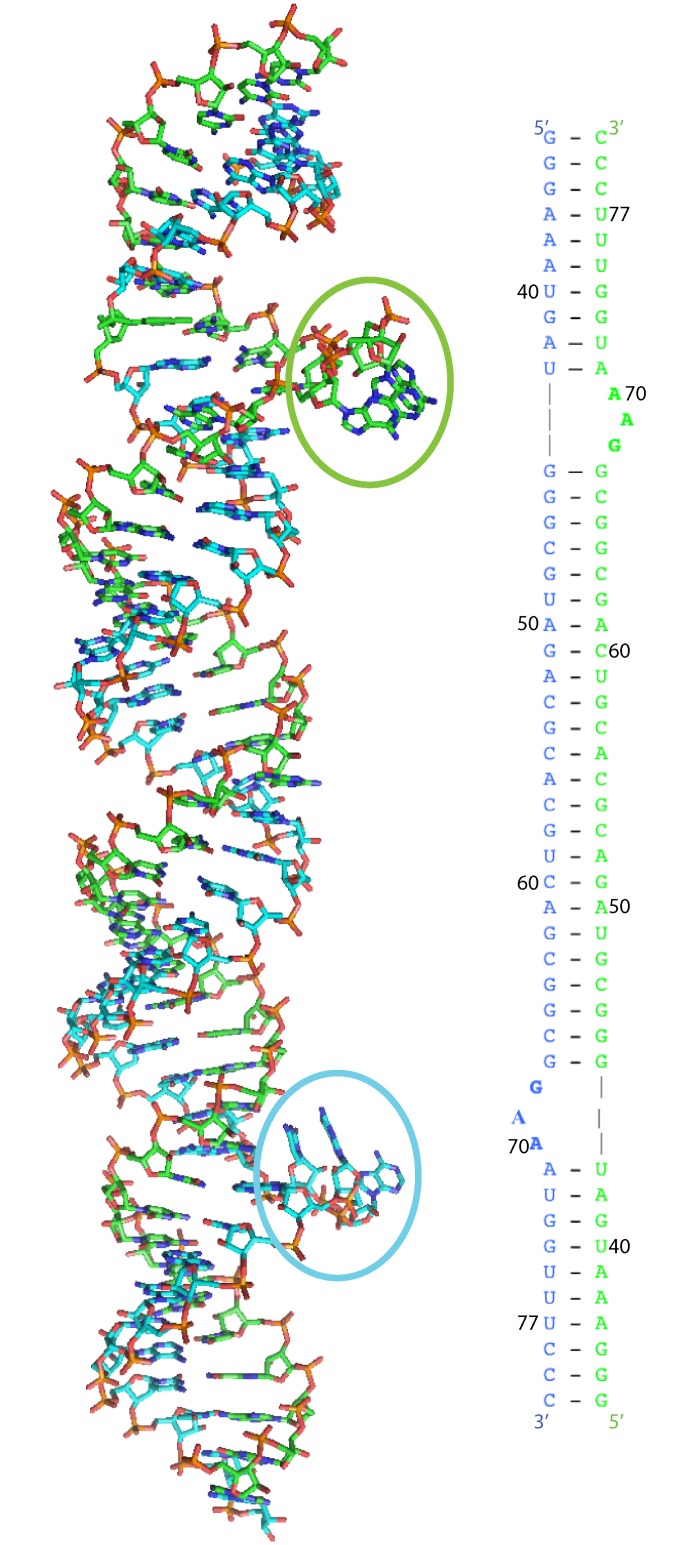
Dimerized structure of 47 nucleotide construct. One chain is shown in green, and the other is shown in cyan. Bases G68 –A70 on the green chain protrude away from the helix, forming a base stacking bulge. The same bases on the cyan chain also protrude from the helix; however, here, G68 and A70 base stack, forcing A69 to further protrude from the helix. The numbering is in accordance with the full-length HAR1 structure.

**Fig 2:**
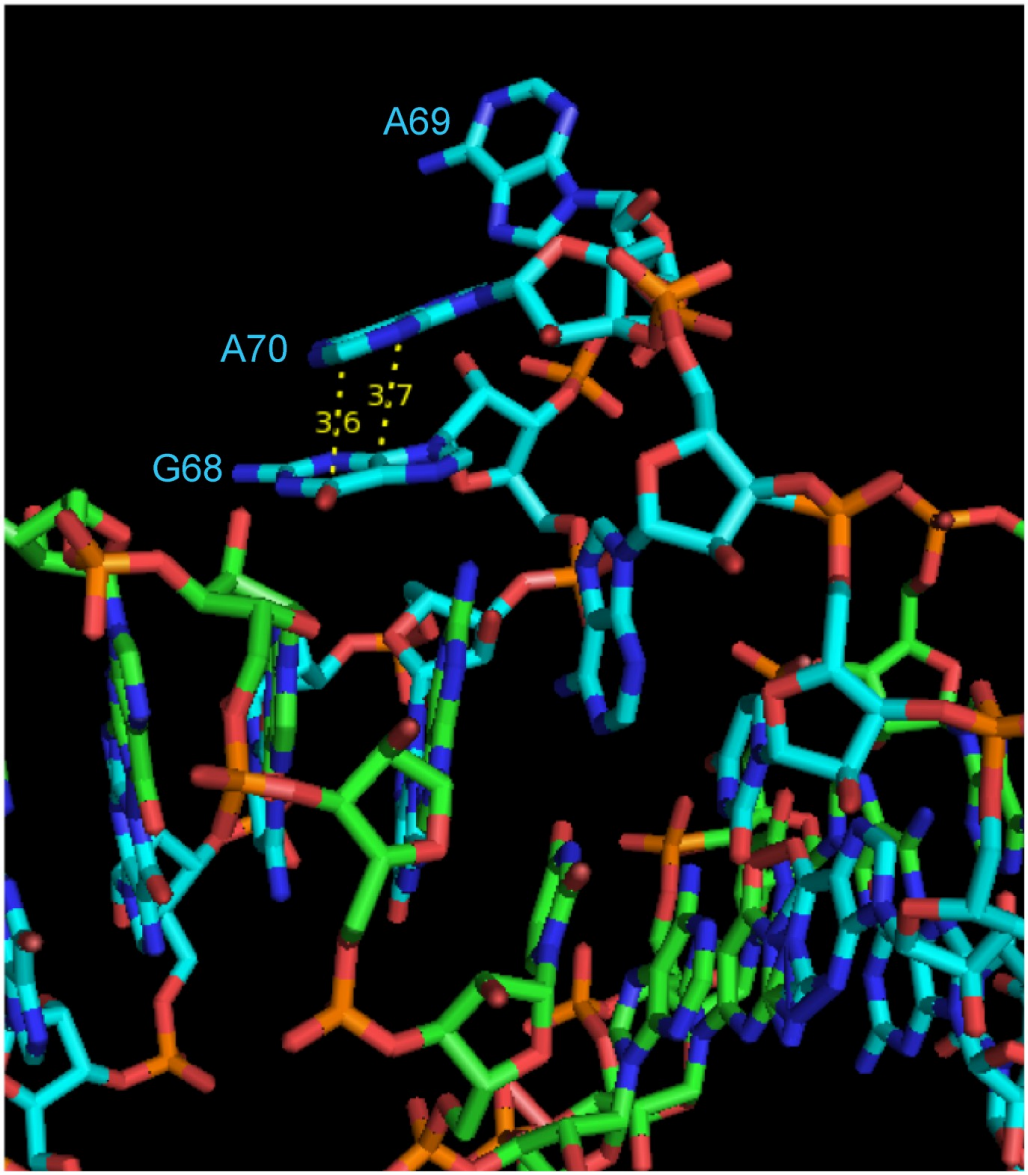
Interaction between G68 and A70 forcing A69 to protrude. The cyan strand showing the first base of the trio, G68, is pointed away from the helix and appears to form a *π*-*π* stacking interaction with A70. This stacking interaction causes base A69 to protrude almost 180° from the helix.

**Fig 3:**
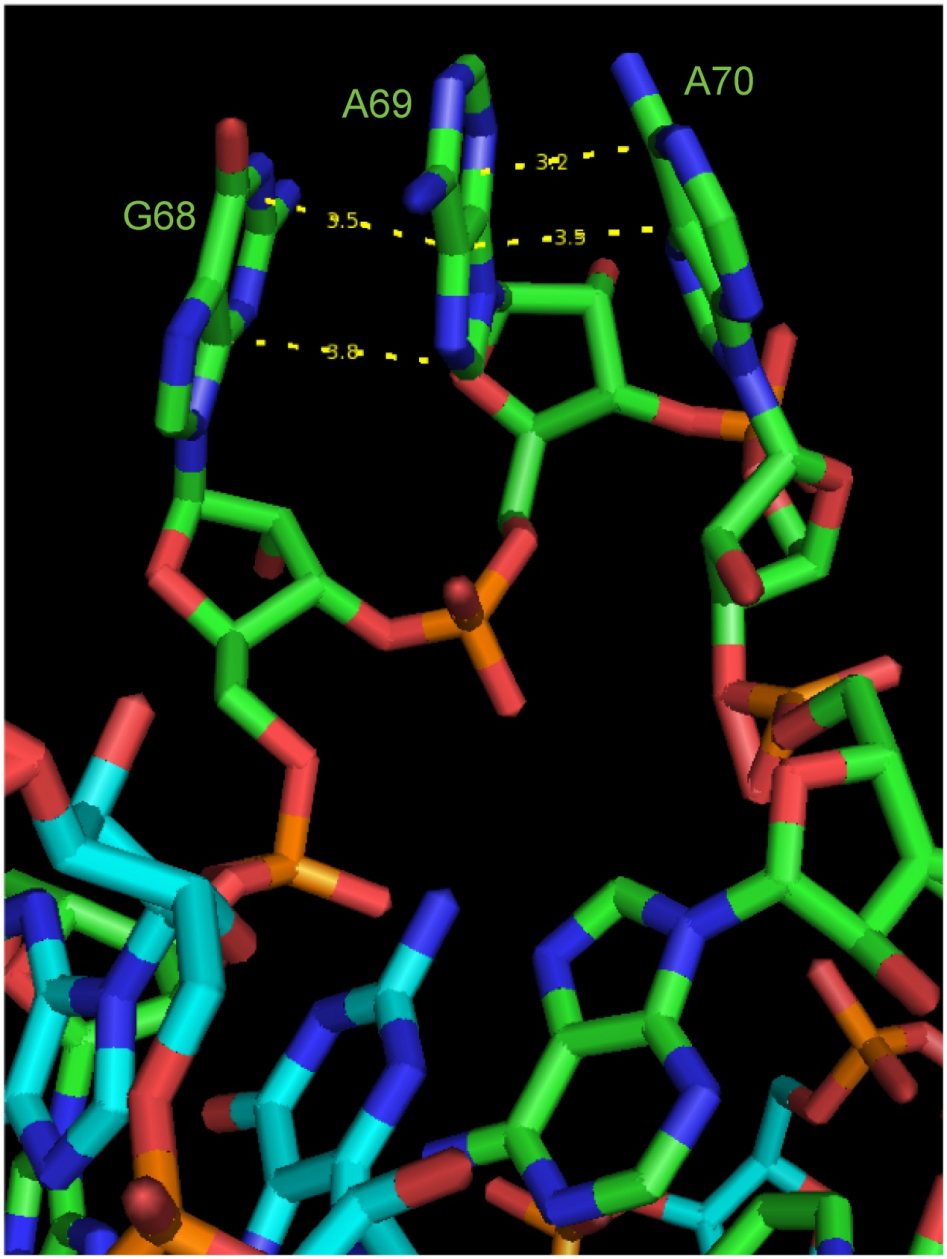
*π*-*π* stacking interaction between G68, A69, and A70. On the green strand, *π*-*π* stacking is observed between G68 and A69. A70 is partially lined up with the base stack; however, the angle of the plane of the base on A70 makes this interaction more tenuous, because its plane is tilted nearly 45° from being parallel to A69.

The crystal formed a linear dimer and showed four complete turns of an RNA helix (Fig 1). The structure showed bulged regions, which were formed on each strand by two extra helical adenines and one extra helical guanine, numbered G68, A69, and A70 in the 118 base construct. On one strand, the first base of the trio, G68, pointed away from the helix and appeared to form a *π*-*π* stacking interaction with A70 (Fig 2). The distance between these two aromatic rings was approximately 3.6 Å, a reasonable distance for this type of interaction. This stacking interaction caused A69 to protrude almost 180° from the helix. On the other strand, a similar *π*-*π* stacking interaction was seen in the bulged nucleotides; however, on this strand, it was between G68 and A69 (Fig 3). A70 was partially lined up with the base stack; however, the angle of the plane of the base on A70 makes this interaction more tenuous, because its plane is tilted nearly 45° from being parallel to A69, thus resulting in an asymmetrical structure.

Structure-probing experiments showed consistency with the predicted secondary structure in the vulnerability of nucleotide A70. Pollard and Beniaminov have indicated base A70 to be vulnerable to methylation by DMS. The bulge formed on each strand of the artificial dimer in this study consists of bases G68, A69, and A70, which are crystallized into two different conformations (Fig 1).

**Fig 4:**
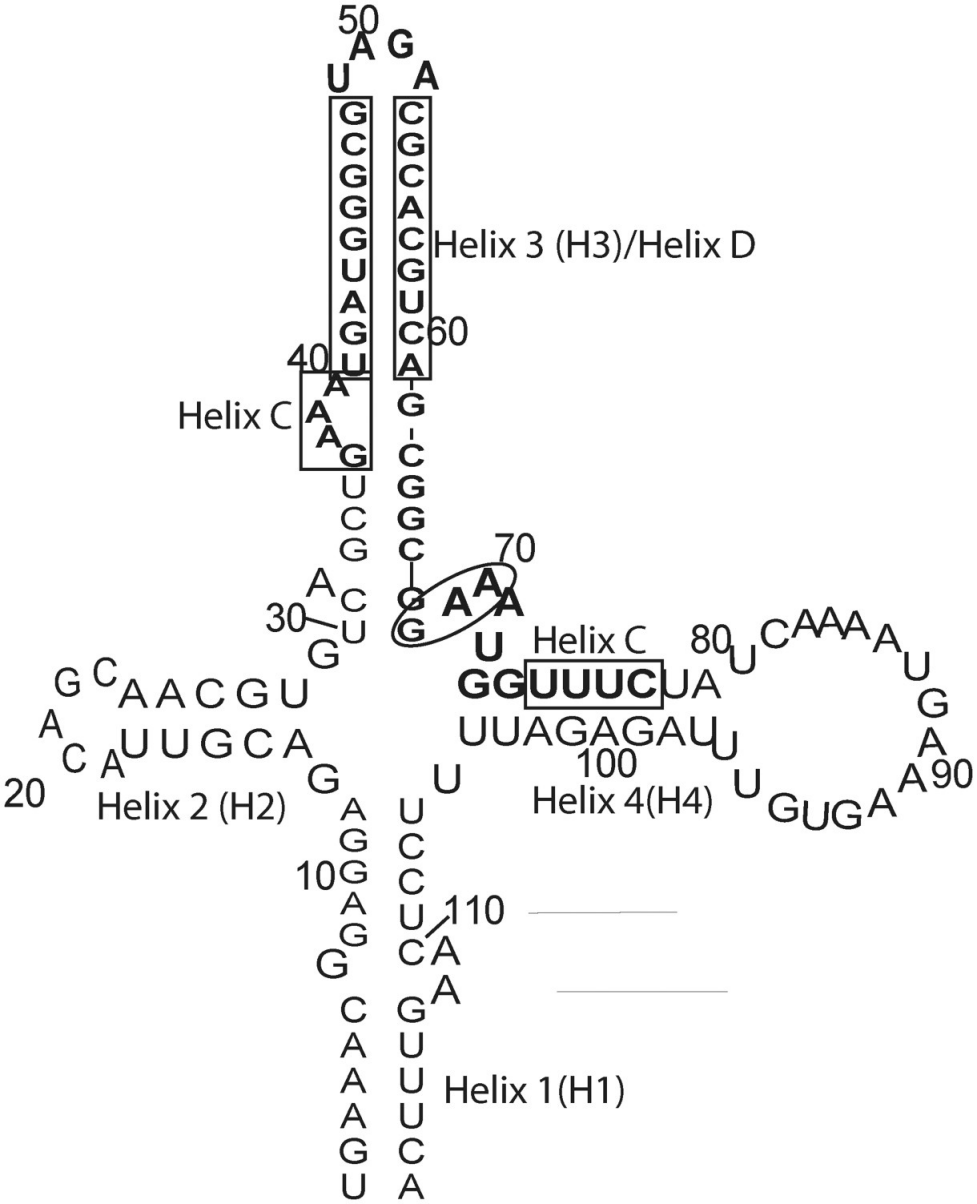
118 nucleotide construct of HAR1. The construct investigated in this study is shown in bold. The three bases enclosed in the oval represent the portion of the structure that formed the bulge. The presented secondary structure is that proposed by Beniaminov. The C and D helices published by Pollard are shown in the boxes.

Beniaminov *et al*. used chemical and enzymatic probing to investigate 12 truncated constructs of HAR1 and designated the helices 1–4. The structure of helix 3, which includes the majority of the C and D helices designated by Pollard, differs from the structure predicted by Pollard (Fig 4). She suggested two helices (C & D) connected through a large loop. This large loop included bases G68—A70. These same bases are still predicted to be single-stranded in Beniaminov *et al*.*'s* structure, just as a smaller bulge (Fig 4). Insights can be gained from the crystal structure reported here because, within the dimer, these bases formed a bulge as predicted by these previous experiments. This suggests HAR1 may interact with putative binding partners through this bulge. The observed bulge is supported by the model of HAR1’s secondary structure predicted by Evofold. The fact that conformational heterogeneity in the bulges was observed is significant because HAR1 may interact with nucleic acid or protein partners. These two conformations provide structural insights about a possible binding site.

Ziegeler *et al*., utilizing a divide-and-conquer method, carried out NMR studies of several smaller constructs of the HAR1 RNA to evaluate the previously proposed secondary structures from Pollard and Beniaminov [5]. These NMR studies also confirmed the helix 3 structure predicted by Beniaminov. The middle junction of the human HAR 1 construct was found to be dynamic as well. Ziegeler *et al*. concluded that the human cloverleaf structure is dynamic, while *in silico* studies suggest that substitutions have led to a more stable human construct [6]. The two different conformations seen in this study suggest that the junction between helices 3 and 4 is dynamic.

The presented structure supports the conclusion that these residues reside in a dynamic unpaired region, as suggested by the NMR studies by Ziegeler *et al*. These three bases are positioned in such a way as to recruit other factors to bind to HAR1. There are examples of an extra helical bulging base acting as a specific site for a protein interaction; frequently, this base is an adenine [14]. An example is in *E*. *coli* ribosomal proteins binding to ribosomal RNA [15]. Another example is that of bacteriophage coat proteins binding to the translational operator of their replicase gene [16]. In this case, a bulged A residue is responsible for a specific RNA-protein interaction.

HAR1 has the potential to give further insight into the biology that makes humans unique and to identify why humans have different cognitive skills, such as working memory, speech, and language. A leading hypothesis is that HAR1 may play a role in aiding neocortex growth, yet the specific function remains unknown. Even with the dimer formation, an inherently dynamic structural motif is present. This motif is observed whether studied by secondary structure prediction, footprinting assays, or NMR. This motif is also present in various lengths of HAR1, as both the full length and smaller constructs have shown this dynamic structural motif. This motif formed no matter what the RNA was binding to or how the RNA was treated. The results of this macromolecular crystal structure led to a nonphysiological dimer, yet this dynamic region is still observed with two different conformations of this structural motif. This suggests that this motif is an inherent property of the HAR1 RNA, and that these protruding bases play a key role in the tertiary interactions HAR1 makes in order to carry out its function.

## Supporting information

S1 Fig Protein data bank validation report (5UNE). Detailed validation report provided by the Proitein Data Bank. The report includes the results of model and experimental data validation for the deposited structure, 5UNE.
(PDF)
